# Relationship between *MspI* polymorphism of *CYP1A1* gene and the risk of endometriosis in an Iranian population: A case-control study

**Published:** 2018-10

**Authors:** Azam Babaki, Ehsan Zare Mehrjardi, Razieh Dehghani Firouzabadi, Abbas Aflatoonian, Seyed Morteza Seifati

**Affiliations:** 1 *Medical Biotechnology Research Center, Ashkezar Branch, Islamic Azad University, Ashkezar, Yazd, Iran.*; 2 *Young Researchers and Elite Club, Ashkezar Branch, Islamic Azad University, Ashkezar, Yazd, Iran.*; 3 *Research and Clinical Center for Infertility, Yazd Reproductive Sciences Institute, Shahid Sadoughi University of Medical Sciences, Yazd, Iran.*

**Keywords:** Endometriosis, Cytochrome P-450 CYP1A1, MspI endonuclease

## Abstract

**Background:**

Endometriosis is a disease that affects women of reproductive age. This disease is characterized by the presence of endometrial-like tissues (endometrial or stromal glands) outside the uterus and shows significantly elevated prevalence in industrial regions. Additionally, an interaction between genetics and environmental factors is assumed for the disease. Enzymes belonging to the cytochrome P450 (CYP) family are participated in detoxiﬁcation process of a wide range of environmental toxins and carcinogens. Thereby, they are good link for the interaction. *CYP1A1* which belong to cytochrome P450 (CYPs) superfamily, is a very important gene for the metabolism of carcinogens.

**Objective:**

The aim of this study was to analyze the frequency of the *MspI *polymorphism of *CYP1A1* gene and its relation to endometriosis.

**Materials and Methods:**

Genomic DNA was isolated from 93 endometriosis women and 139 healthy controls. Genotyping was performed using polymerase chain reaction followed by restriction fragment length polymorphism analysis.

**Results:**

Frequencies of the TT, TC, and CC genotype of *CYP1A1* gene polymorphism in patients were 73.1%, 22.6%, and 4.3%, while frequencies in controls were 74.1%, 22.3%, and 3.6%, respectively. So there was no significant differences between the genotypes in two groups (p=0.961).

**Conclusion:**

According to our study, *MspI* polymorphism of *CYP1A1* gene appears to be not associated with the risk of endometriosis in the studied population. However, additional studies, especially with larger sample size are needed to validate these findings.

## Introduction

Endometriosis is defined as the presence of endometrial glands and stroma outside their normal locations (1, 2). This disease is one of the main causes of infertility and chronic disorders among women, affecting approximately 7-10% of women in their fertility age and 5-50% of infertile women (3-6). It has a wide range of symptoms of which pelvic pain, dysmenorrhea, dyspareunia, and abnormal uterus bleeding are the most important ones (7). Endometriosis is a complex trait. Some of studies have suggested the role of immunological, endocrine, environmental and genetics factors in the pathogenesis of this disease. Moreover, it has been revealed that endometriosis is more common in first degree relatives of endometriosis patients compared to first degree relatives of unaffected individuals; reflecting the involvement of genetics factors (3-5). Increasing evidences have shown that multiple gene loci interact with each other and with the environment to produce endometriosis (6, 7). So far, the relationship between endometriosis and different genes has been investigated. Among them, genes involved in detoxification systems such as Cytochrome (*CYP*) P450 superfamily are the most important ones and possibly a good link for interactions of genetic and environmental factors (8, 9). Enzymes of this family are participated in detoxification process of a wide range of environmental pollutants and carcinogens (10).

*CYP1A1*, a member of P450 cytochrome superfamily, is located on chromosome 15 (15q 24.1) (11). *CYP1A1* rs4646903 (T>C) polymorphism, downstream from the polyadenylation site at the 3´ end of exon 7, is one of the most important polymorphisms of the gene. Substitution of T to C at this position formed a target site for *MspI* enzyme, so this polymorphism is also named *MspI* or M1 (11, 12). It has been reported that this polymorphism is correlated with CYP1A1 enzyme inducibility phenotype. There are some studies reported the relationship between *MspI* polymorphism and the risk of endometriosis (13, 14). 

The goal of this study was investigation of the association between *MspI* polymorphism of *CYP1A1* gene and endometriosis in women referred to two infertility centers in Yazd, Iran who were from central and southern parts of Iran.

## Materials and methods

In this case-control study, 93 women with endometriosis (age range 20-42 yr) referred to two infertility centers in Yazd, Iran: Madar Hospital and Yazd Research and Clinical Center for Infertility were enrolled in this study as the case group. Endometriosis was diagnosed by an experienced gynecologist through laparoscopy. Guidelines of American Society for Reproductive Medicine (ASRM) was used to classify patients (ASRM) into 4 stages; minimal (stage I), mild (stage II), moderate (stage III), and severe (stage IV) (15). 

The control group consisted of 139 unrelated healthy premenopausal women (age range: 18-50 yr) undergoing Cesarean section or hysterectomy at the same centers with no history of endometriosis and without any lesion suggesting endometriosis during Cesarean section or hysterectomy. Women with a history of autoimmune disease and cancer were excluded from the research. Clinical characteristics of all the participants were collected personally using a designed questionnaire. 

**Genotype determination**

Genomic DNA was extracted from 5 mL of EDTA anticoagulated whole blood using AccuPrep® Genomic DNA Extraction Kit (Bioneer, South Korea) based on manufacturer's manual. To study *MspI* polymorphism of *CYP1A1* gene, PCR-based restriction fragment length polymorphism (RFLP) method was used. The specific primers for the target region (forward primer of 5'-TAGGAGTCTTGTCTCATGCC-3' and reverse primer of 5'-CAGTGAAGAGGTGT AGCCGC-3') was designed by Gene Runner (version 5.1) software and synthesized by Macrogen (South Korea). PCR reaction mixture was contained 1X PCR buffer, 2 mM MgCl_2_, 2.5 unit of Taq DNA Polymerase (Cinnagen, Iran), 0.5 mM dNTP, 5 pM of each primer, and 30 ng of pattern DNA up to the volume of 25 μl of sterile distilled water. Ampliﬁcation was performed as follows: initial denaturation at 95^o^C for 5 min, then 30 cycles of denaturation at 94^o^C for 30 s, annealing at 60^o^C for 45 s, extension at 72^o^C for 60 s and final extension at 72^o^C for 10 min.

The ampliﬁcation products (343 bp) were digested with *MspI* (Fermentas, Germany) for 16 hr at 37ºC. Then *MspI* restriction digestion products were loaded on 2% agarose gel. The genotypes were characterized as TT when the PCR products remained uncut (343 bp), as CC when the restriction site was present, and as TC when the digestion produced a pattern of three DNA fragments sized 343 bp, 136 bp, and 207 bp.

**Ethical consideration**

The Islamic Azad University, Yazd Branch Ethics Committee approved this study (IR.IAU.YAZD.REC.1396,23). Written informed consent was obtained from all participants. 

**Statistical analysis**

The Pearson χ^2^ or Fisher's exact test was used to compare genotype distributions between patients and controls. The statistical modeling using logistic regression was used to calculate the relative risk (odds ratio; OR) of genotypes for case-control study. Odds ratios were expressed together with the 95% conﬁdence interval (16). p≤0.05 was considered statistically signiﬁcant. The Hardy-Weinberg equilibrium analysis was conducted by comparing observed versus expected genotype frequencies using a Chi-square test.

## Results

The mean age of the case group was 28.91±5.18 yr and 29.85±8.77 yr in control group (p=0.356). The mean body mass index (BMI) was 24.30±3.69 in the case group and 26.14±4.82 in controls. Statistical analysis showed that BMI of the cases is significantly less than control group (p=0.003) (Table I). Stage II of the disease has the highest frequency (40.20%). Totally, 51 patients (55.40%) had primary stages of disease (I, II) and 41 of them (44.60%) were in the advanced stages of endometriosis (III, IV). Regarding both controls and patients; the distribution of *MspI* genotypes was in Hardy-Weinberg equilibrium. 

The most frequent genotype in two groups was TT. The frequencies of this genotype in patients and control group were 73.1% and 74.1%, respectively. Data analysis by χ^2^ test revealed that there is no significant difference between the two groups (p=0.961). Using the TT homozygote as a reference group, the OR of TC heterozygotes (OR, 1.016; 95% CI, 0.54 to 1.93) and CC homozygotes was (OR, 1.204; 95% CI, 0.31-4.6). Allele frequencies of *MspI* polymorphism in the case and control groups are shown in table II. Comparison between the alleles by χ^2^ test indicates no significant difference between two groups (p=0.804). In all stages of endometriosis, CC genotype has the lowest frequency of 7.2%, 5.4%, 0.0%, and 4.5% for stages I to IV, respectively (Table III). Statistical analysis of the frequencies of *MspI* genotypes by Fisher's exact test indicated that there is no significant difference between the genotypes and different stages of endometriosis (p=0.491). In addition, the patients with advanced stages were compared with the primary stages and control group; there was no significant difference between two groups (p=0.427 and p=0.507, respectively).

**Table I:** General characteristics of the case and control groups

**Characteristic**	**Case**	**Control**	**p-value**
Age (yr)*	28.91 ± 5.18	29.85 ± 8.77	0.356 a
BMI (kg/m2)*	24.09 ± 3.70	26.14 ± 4.82	0.003 a
Primary infertility**	64 (76.2)	7 (63.6)	0.373 b
Secondary infertility**	20 (23.8)	4 (36.4)	

p>0.05 was considered statistically significant.

* Data presented as mean±SD.

a Students’ *t* test

** Data presented as n (%).

b Fisher's exact test

**Table II:** Genotype and allele frequencies of *MspI* polymorphism in the caseand control groups

**Genotypes and allele**	**Case group**	**Control group**	**Odds ratio**	**p-value**
CC	4 (4.3)	5 (3.6)	1.204 (0.31-4.6)	0.961^a^
TC	21 (22.6)	31 (22.3)	1.016 (0.54-1.93)	
TT	68 (73.1)	103 (74.1)	Ref.	
T	157 (84.4)	237 (85.3)	Ref.	0.804^b^
C	29 (15.6)	41 (14.7)	0.9366 (0.5587-1.5699)	

Data presented as n (%). p>0.05 was considered statistically significant.

a Fisher's exact test.

b χ^2^ test

**Table III:** Genotype frequencies of *MspI* polymorphism in different stages of endometriosis

** Severity**	**Minimal **	**Mild **	**Moderate**	**Severe**	**Total**
**Genotype**					
TT	10 (71.1)	23 (62.2)	17 (89.5)	17 (77.3)	67 (72.8)
TC	3 (21.4)	12 (32.4)	2 (10.5)	4 (18.2)	21 (22.8)
CC	1 (7.2)	2 (5.4)	0 (0)	1 (4.5)	4 (4.4)
Total	14 (100)	37 (100)	19 (100)	22 (100)	92 (100)

Data presented as n (%). p>0.05 was considered statistically significant. Fisher's exact test (p=0.491)

## Discussion

The role of products of *CYP1A1* gene in metabolism of estrogen and PAH has been well-defined (9, 17). It was observed that *CYP1A1* was implicated in metabolism of dioxin, which may increase the severity of endometriosis in rhesus monkeys (18). Therefore, considering that endometriosis is an estrogen-dependent disease (19), *CYP1A1* may be associated with this disease. The level of gene expression or mRNA stability can be altered by the *CYP1A1* *MspI* polymorphism which resulting in a highly inducible activity of the enzyme (20).

In the present study, results showed that there is no statistical significant difference between the frequencies of the genotypes in two groups (p=0.961). Previous reports on association of *MspI* polymorphism of *CYP1A1* gene with endometriosis are controversial. In consistency with our findings, there are some studies in different populations such as in UK (p=0.11) (15), China (p>0.05) (21), India (p>0.05) (22, 23), Austria (p=0.6) (24), Taiwan (p>0.05) (1), USA (p=0.65) (14) and Brazil (p=0.087) (12), which did not found any correlation between the polymorphism and endometriosis. In contrast, Arvanitis and colleagues examined Greek population and noticed that TT genotype will result in 38% decrease in endometriosis risk (OR, 0.62; 95% CI, 0.440 to 0.883) (13). In our study, there was no significant difference between the allele frequencies of two groups (p=0.804). The same results were reported by some other studies (1, 14, 15, 21-24). In contrast, Barbosa and colleagues suggested that the frequencies of C allele was higher in Brazilian women with endometriosis as compared to the control group (p=0.006). Diversity in genetics and environmental background, sample size and selection of control group might be reason for differences among results. In our research, no significant difference was observed between age and cause of infertility of patient and control groups. However, mean BMI of the patients was less than the control group and this difference was statistically significant (p=0.003). These findings confirmed the results of other studies which were reported decrease of BMI is associated with endometriosis (1, 14, 25, 26).

## Conclusion

In conclusion, this study showed that *CYP1A1 MspI* polymorphism was not associated with endometriosis risk in our population. More studies with larger sample size and especially, in other populations with different ethnicity can provide a better understanding of the relationship between population-specific environmental variables and genetic backgrounds.

## Note

***This article extracted from M.Sc thesis. (Azam Babaki)***
